# Carcinogenic effect of arsenic in digestive cancers: a systematic review

**DOI:** 10.1186/s12940-023-00988-7

**Published:** 2023-04-17

**Authors:** Sophie Kasmi, Laureline Moser, Stéphanie Gonvers, Olivier Dormond, Nicolas Demartines, Ismail Labgaa

**Affiliations:** 1 Division of Internal Medicine, Lausanne University Hospital (CHUV), University of Lausanne (UNIL), Lausanne, Switzerland; 2 Division of Gynecology, Lausanne University Hospital (CHUV), University of Lausanne (UNIL), Lausanne, Switzerland; 3 Division of Visceral Surgery, Lausanne University Hospital (CHUV), University of Lausanne (UNIL), Rue du Bugnon 46, CH-1011 Lausanne, Switzerland

**Keywords:** Oncogenic, Toxicants, Heavy metals, Carcinogenesis, Prevention

## Abstract

**Background:**

The carcinogenic effect of arsenic (As) has been documented in lung, bladder and skin cancers but remains unclear for digestive cancers, although metabolic pathways of As and recent data suggest that it may be an important determinant in these malignancies as well.

**Objective:**

This study aimed to systematically review the available literature investigating the potential association between As and digestive cancers.

**Methods:**

An extensive search was conducted in Medline Ovid SP, Cochrane, PubMed, Embase.com, Cochrane Library Wiley, Web of Science and Google Scholar. Studies providing original data in humans, with As measurement and analysis of association with digestive cancers including esogastric cancers (esophagus and stomach), hepato-pancreatico-biliary (HPB) cancers (including biliary tract, liver and pancreas) and colorectal cancers were eligible.

**Results:**

A total of 35 studies were identified, 17 ecological, 13 case–control and 5 cohort studies. Associations between As and digestive cancers were reported for both risks of incidence and cancer-related mortality. Overall, 43% (3/7) and 48% (10/21) studies highlighted an association between As and the incidence or the mortality of digestive cancers, respectively.

**Conclusions:**

A substantial proportion of studies exploring the potential link between As and digestive cancers suggested an association, particularly in HPB malignancies. These findings emphasize the need to further investigate this topic with dedicated and high-quality studies, as it may have an important impact, including for prevention strategies.

**Supplementary Information:**

The online version contains supplementary material available at 10.1186/s12940-023-00988-7.

## Introduction

Digestive malignancies represent a major health problem. In 2018, 4.8 million of new cases and 3.4 million of deaths were reported, worldwide (esophagus, stomach, colorectal, liver and pancreatic cancers) [1]. An increasing burden related to these cancers is predicted [2]. Therefore, it is paramount to better understand their etiology and identify risk factors associated with each of these malignancies [3].

Arsenic (As) is a heavy metal and component of the earth crust that may contaminate water, air, soil and food. Exposure to arsenic in humans occurs mainly through contaminated subsoil water, industrial exposure, food and tobacco. Metabolized by the liver, its metabolites are excreted through bile -for the most toxic ones- and urine [4]. The magnitude of excessive exposure to As seems very important, with 108 countries affected by As-contaminated drinking water, translating to 40 million individuals exposed to As concentrations above the limit established by the World Health Organization (WHO) [5].

The geographical distribution of As is heterogeneous, with areas like Chile, India or Bangladesh showing particularly high concentrations associated with devastating repercussions. As an illustration, the WHO estimated that the As crisis in Bangladesh was the “*largest mass poisoning of a population in history*” [6]. The carcinogenic effect of As has been demonstrated in bladder, lung and skin cancers, while the evidence is limited for liver, biliary, kidney and prostate cancers [7].

The role of As in digestive malignancies is not clear. Some recent studies suggest a carcinogenic effect of As in biliary cancers [8-10]; however there is a lack of data for the mechanism by which this occurs. The paucity of data is surprising considering that As most toxic metabolites are excreted in the bile [11] and therefore the occurrence of biliary and digestive cancers could be expected.

The exact carcinogenic mechanisms of arsenic in humans is not fully elucidated but several elements have been postulated, including cell damage induced by the generation of reactive oxygen species and nitrogen species, genotoxic damage induced by As and signaling pathways activation/inhibition related to gene expression variations [12, 13].

This study aimed to systematically review the available data on the association between As and digestive cancers.

## Materials and methods

This systematic review was conducted according to the recommendations of the Cochrane Handbook for Systematic Reviews and Interventions [14] and according to PRISMA guidelines [15]. A protocol of the review was established a priori and registered in the PROSPERO database (CRD42022348424).

### Search strategy

An extensive review of the literature was conducted to identify studies investigating the association between As and digestive cancers. The following databases were queried until February 28, 2022: Medline Ovid SP, Cochrane, PubMed, Embase.com, Cochrane Library Wiley, and Web of Science (Core Collection). A supplementary search has been conducted in Google Scholar, Embase, Ovid SP, Wiley and Web of Science. All searches were conducted without language or date restrictions. Algorithms of search with specific syntaxes for each database are detailed in Additional File 1. Cross-referencing (searching the reference lists of the included studies) was also performed to identify studies that might have not been identified during the initial search.

### Study selection, data extraction and quality assessment

Two investigators (S.K. and L.M.) independently applied the selection criteria provided in Table 1. In case of disagreement, a third investigator (I.L.) took a consensus decision. The following variables were extracted from each selected study: design, country, number of subjects, source of As exposure and main findings. Quality of selected studies was assessed according to the National Toxicology Program handbook for preparing report on carcinogens monographs [16] (Additional File 2). Of note, quality assessment was not used as a selection criteria.

**Table 1:** Criteria for study selection

Study selection
• Human data (in vitro and in vivo studies were excluded)
• Original data (reviews, commentaries, editorials, systematic review and meta-analyses were excluded)
• Measurement of arsenic biomarkers (studies measuring arsenic in samples such as air, water, soil or any other source)
• Studies on the association between arsenic and digestive cancers, providing details about the origin of the primary cancer
• The most recent and complete article was chosen if a study had been published more than once
• Available full-text publications in English

## Results

**Fig. 1:**
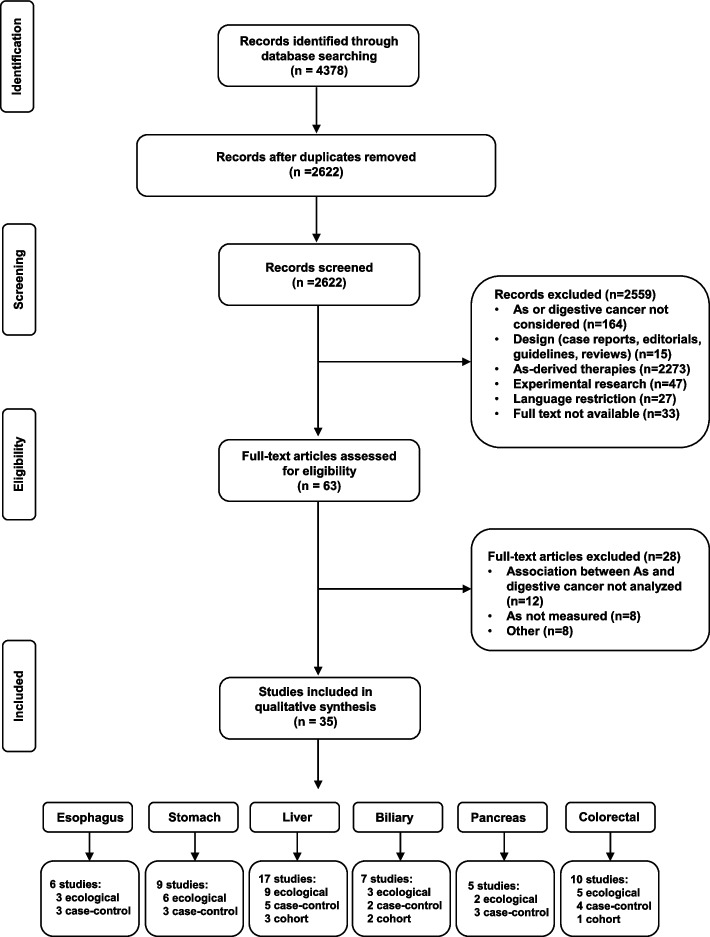
Flowchart. Study selection process. Abbreviations: As: arsenic

A comprehensive search of the literature identified 2622 studies after removal of duplicates (Fig. 1). A thorough screening reduced this number to 63 potentially eligible reports. Twenty-eight studies were excluded based on selection criteria, leading to the final inclusion of 35 studies. These included 17 ecological, 13 case–control and 5 cohort studies. Thereafter, studies were categorized based on the organ of the primary tumor: esophagus (*n* = 6), stomach (*n* = 9), biliary (*n* = 7), liver (*n* = 17), pancreas (*n* = 5) and colorectal (*n* = 10). Of note, 8 studies investigated different types of digestive cancers in the same report. Relevant findings generated by the selected studies will be detailed for each organ.

### Esogastric cancers (ICD codes C15 and C16)

#### Esophagus (ICD code C15)

Six studies were selected (Additional file 3). These included 3 ecological and 3 case–control studies, essentially conducted in As-endemic regions such as countries of the Middle East.

A Spanish ecological study investigated the potential association of topsoil toxic metals on various cancer-related mortalities, including esophageal cancer (EC) [17]. A variety of heavy metals were measured and the authors utilized relatively sophisticated mathematical models to infer mortality in a dataset covering 861,440 cancer deaths, according to 13,317 topsoil samples measuring heavy metals. A total of 14,287 EC deaths were included in the analysis. While no association of As was detected on EC mortality, with an insignificant risk ratio (RR) in men (RR: 1.02, 95% CI: 0.96–1.08), a marginally significant protective effect was calculated in women (RR: 0.89, 95% CI: 0.80–1.00).

Based on previous findings identifying nickel (Ni) and As as risk factors for oral cancers, Lee et al. launched a subsequent study on Ni and As in EC, showing an association between EC prevalence and Ni but not As [18]. Blackfoot-disease (BFD), a dermatosis that is pathognomonic of an excessive exposure to As, is endemic in certain areas of Southeast Asia like Taiwan. Comparison of mortality due to cancer and non-cancer diseases were performed between BFD-endemic areas of Taiwan with local and national reference groups in men and women [19]. A gender-dependent effect of As on EC mortality was observed, showing a significantly higher standardized mortality ratio (SMR) in men (SMR: 1.67, 95% CI: 1.30–2.12) but not in women (SMR: 1.58, 95% CI: 0.82–2.76).

In a case–control study conducted in Pakistan, investigators used hair samples as a proxy of overall exposure to heavy metals including As in a cohort of patients with various types of cancers. EC patients showed higher levels of As, as opposed to healthy controls [20].

A recent cohort study conducted in Iran measured As in EC and non-cancer tissue samples showing comparable median concentrations of 0.6 and 0.8 µg/kg, respectively (*p* = 0.328) [21].

#### Stomach (ICD code C16)

Nine reports investigated the consequences of As on gastric cancer (GC), including 6 ecological and 3 case–control studies. López-Abente et al. found no association between As and GC [17], conversely to EC, where a marginally protective effect of As was detected on mortality in women,

Likewise, a Japanese study enrolling a large cohort of patients identified no association between air concentration of As and GC-related SMR [22]. Chen et al. analyzed the effects of As in soil, in the region of Suzhou (China) on age-adjusted mortality related to a number of health conditions including some digestive cancers (stomach, liver and colon) [23]. Regarding GC, they first demonstrated a positive and significant correlation (Spearman = 0.412, *p* < 0.01). Furthermore, the quasi-Poisson regressions for the effects of As and Ni in soils showed significantly increased RR in men (RR: 1.114, 95% CI: 1.063–1.168, *p* < 0.001) and in women (RR: 1.105, 95% CI 1.051–1.161, *p* < 0.001).

Kohzadi et al. performed inductively coupled plasma mass spectrometry (ICP-MS) to measure concentrations of several heavy metals in tissue [24]. Therewith, they measured As in 35 GC patients in cancer and non-cancer (adjacent to the tumor) tissues, as well as in samples from 30 controls. The authors reported higher As levels in GC tissue, compared to controls.

In 2013, an ecological study conducted in Ireland aimed to investigate the relationship between trace elements in soil and cancer incidence, including GC [25]. Correlations widely varied according to the regions, showing a heterogeneous distribution, with areas displaying high correlations (coefficient = 0.69) and other regions showing low correlations (coefficient = -0.02).

Like most South American countries, Argentina also encompasses regions with high concentrations of As in drinking water. Its association with SMR related to GC was evaluated in Córdoba, showing insignificant results (SMR in women: 1.04, 95% CI: 0.87–1.22) [26].

In Taiwan, mortality related to GC appeared higher in BFD areas, both in men (SMR: 1.36, 95% CI 1.17–1.46) and women (SMR: 1.40, 95% CI 1.15–1.68) as compared to control regions [19].

Nozadi et al. also utilized ICP-MS to measure As in tissues samples of GC patients compared to controls but did not find any difference [21].

### Hepato-pancreatico-biliary cancers (ICD codes C22, C23, C24 and C25)

A total of 25 articles investigating hepato-pancreatico-biliary (HPB) cancers were selected (Additional file 4).

#### Liver (ICD code C22)

Liver cancer (LC) was the most frequently investigated digestive malignancy, with 17 studies identified: 9 ecological, 5 case–control and 3 cohort studies.

Five studies were conducted in Taiwan, including 3 analyses of BFD regions. Tsai et al. also analyzed cancer mortality related to hepatocellular carcinoma (HCC) in BFD areas and found that LC mortality was higher in men (SMR 1.83, 95% CI 1.69–1.98) and women (SMR 1.87, 95% CI 1.64–2.14) compared to control regions [19].

Chen et al. reported similar findings [27] whereas the study by Guo et al. did not detect any difference between BFD and control regions [28].

Lin et al. analyzed As concentrations and LC-related mortality and showed that As concentrations in drinking water > 0.64 mg/L were associated with higher LC-related mortality. Conversely, no association? was detected for low As concentrations (< 0.64 mg/L) [29]. A study in Argentina also showed increased SMR in high-level As exposure areas [26].

In 1955, the western part of Japan suffered from a mass As poisoning due to contaminated milk powder. A study hypothesized that this event may have been associated with long-term injuries in the exposed population, including digestive cancers (liver and pancreas) [30]. The authors compared cancer-related mortality of these individuals with that of non-exposed controls. Regarding liver cancer (LC), the exposed cohort showed higher mortality (MR 1.73, 95% CI 1.31–2.28).

Also tackling the question of early-life exposure, Smith et al. leveraged the unique epidemiological scenario described in the city of Antofagasta in Chile where the population was exposed to very high concentrations of As in drinking water (870 µg/L) in 1958, with an abrupt stop in 1970 [31]. This natural intervention allowed estimation of health effects attributed to As exposure. LC-related mortality of 30–49-year-old persons between 1989–2000 was increased in this area as compared to the rest of the country (SMR: 2.5; 95% CI: 1.6–3.7).

In 2008, Baastrup et al. published a study with prospectively collected data [32]. They used a cohort of 53,053 individuals from two Danish areas and linked cancer cases from the Danish Cancer Registry with geocoded residential addresses. This methodology allowed to analyze the association of low As levels in drinking water (mean 1.2 µg/L). No significant association between As exposure and LC incidence was found (RR 0.89, 95% CI: 0.73–1.08, *p* = 0.24).

As concentrations in blood of 314 patients with HCC were found to be significantly higher as compared to control subjects (0.237 ± 0.117 *vs.* 0.019 ± 0.008 mg/L, *p* < 0.001), in a case–control study conducted in Egypt [33].

Cano et al. analyzed As concentrations in tumor and non-tumor tissue samples of 76 patients with HCC in a non-cirrhotic liver in Peru and France and found higher As levels in the Peruvian cohort, both in tumor and non-tumor samples [34].

Another cohort study conducted in Bangladesh revealed a lifetime excess risk of LC-related mortality attributable to As in drinking water of 0.9 in men and 3.4 in women, per 100,000 population [35].

Hsu et al. evaluated the interaction between arsenic exposure and HBV or CV infection in chronic liver disease [36]. Among seropositive participants, exposure to high-arsenic drinking water (≥ 100.0 μg/L) was associated with a reduced risk of liver cancer (HR, 0.29; 95% CI, 0.09–0.95; *p* < 0.05).

#### Biliary tract (ICD codes C23 and C24)

A total of 7 articles were identified, with 6 studies on gallbladder (GBC) and one on bile duct cancer (BDC). It included 3 ecological, 2 case–control and 2 cohort studies.

Tsai et al. analyzed cancer mortality related to GBC but found no effect of As [19].

In 2020, Ganesan et al. specifically explored the effect of As-contaminated water on GBC incidence [8]. First, a positive correlation was noted in women worldwide (Spearman = 0.31, *p* = 0.03). These findings were confirmed by national-scale analyses in the US, Taiwan and India. The same group performed a study in BDC, showing similar results [9].

A study conducted in India aimed to assess the geographical pattern of GBC according to the proximity to River Ganga, a widely known source of heavy metal intoxication [37]. Odds ratios (OR) of districts along River Ganga (OR 1.72, 95% CI: 1.54–1.91, *p* = 0.001) and those with high concentrations of As in soil (OR 1.45, 95% CI: 1.30–1.62, *p* = 0.001) showed higher risk of GBC.

A recent study performed metallomic analyses in serum with a panel of 18 metals in a large cohort of patients including 259 with GBC, 701 with gallstones and 851 controls [38]. Surprisingly, patients with GBC showed lower As levels than patients with gallstones, and controls displayed the highest levels of As in serum. Furthermore, As levels were inversely associated with the risk of GBC, when comparing the lowest tertile, T1, to T2 (OR 0.38, 95% CI 0.26–0.55, *p* < 0.001) and T3 (OR 0.20, 95% CI 0.13–0.29, *p* < 0.001). Intrigued by these results, a European group of researchers utilized Mendelian randomization analysis to decipher the association of As [39]. In contrast to the former study that only assessed total As, this consortium analyzed the various As species, namely inorganic As (iAs), monomethylarsonic acid (MMA) and dimethylarsonic acid (DMA). Integrating genomic factors such as arsenite methyltransferase gene (*AS3MT*) variants permitted to distinguish the impact of each As species. While the data confirmed a protective effect of iAs and MMA, it highlighted a deleterious effect of DMA on the risk of GBC.

Finally, Kumar et al. detected a significant correlation between age and As levels in blood samples from 175 patients with GBC (*r* = 0.005, *p* < 0.05), in a case–control study [40].

#### Pancreas (ICD code C25)

Five articles explored the association between As exposure and pancreatic cancer (PC), including 2 ecological and 3 case–control studies.

An ecological study conducted in Taiwan showed comparable mortality related to PC between BFD endemic areas and control regions, in men (SMR 1.22, 95% CI: 0.82–1.74) and women (SMR: 0.96, 95% CI: 0.58–1.50) [19].

The Japanese case–control study investigating the As-mass poisoning in milk powder also analyzed PC-related mortality [30]. Results were very similar to LC, with a higher mortality related to PC reported in the exposed group (MR 1.79, 95% CI: 1.23–2.61).

A Spanish consortium took advantage of the multicentric cohort PANKRAS II [41] to assess the relation between trace elements and exocrine pancreatic cancer (EPC). The cohort included 118 EPC and 399 healthy controls in whom trace elements including As were measured in toenails [42]. Patients of the highest quartile for As level (> 0.1061 µg/g) were at higher risk to develop EPC (OR: 2.02, 95% CI: 1.08–3.78, *p* = 0.009). In a follow-up study, the authors aimed at deciphering a potential link between these trace elements and oncogenic driver mutations in *KRAS* [43]. Sample size was reduced, with 78 patients harboring pancreatic ductal adenocarcinoma (PDAC) and 416 controls. The authors did not show an association of As on PDAC (aOR: 1.62, 95% CI: 0.95–2.78). Subgroup analysis showed neither an association between As and wild-type KRAS (aOR: 3.37, 95% CI: 0.98–11.57) nor with mutant KRAS PDAC (aOR: 1.73, 95% CI: 0.85–3.53).

### Colorectal cancer (ICD codes C18, C19, C20 and C21)

Additional file 5 details 10 selected articles, including 5 ecological, 4 case–control and 1 cohort studies.

The previously cited Danish study also investigated the effect of low As levels in drinking water on colon cancer incidence, showing no significant association (IRR: 0.97, 95% CI: 0.93–1.01, *p* = 0.1) [32]. Despite the prospective nature of the data, these populations were exposed to low levels of As.

The incidence of colon cancer was monitored in Córdoba (Argentina), a region known for high concentrations of As in water. A gender-specific effect of As was detected, showing a detrimental impact in women (IRR: 12.21, 95% CI: 5.72–26.07, *p* < 0.01) but a protective effect in men (IRR: 0.03, 95% CI: 0.02–0.06, *p* < 0.01) [44].

The Taiwanese ecological study by Tsai et al., mentioned above for esogastric and HPB cancers, also analyzed the effect of As in BFD endemic areas for colorectal cancer (CRC)-related mortality [19]. A significant association was detected for colon cancer, with an SMR reaching 1.49 (95% CI: 1.20–1.83) in men and 1.42 (95% CI: 1.13–1.76) in women. The study also provided rare data on cancers of the small intestine, showing no significance in women (SMR: 1.38, 95% CI: 0.59–2.72) but highlighting As as a determinant in men (SMR: 7.15, 95% CI: 1.20–3.54).

In the early 1960s, Taiwan implemented a tap water supply system in BFD-endemic areas to prevent As-induced deleterious effects. Yang et al. aimed at analyzed the effect of this measure on CRC-related SMR between 1971 and 2006 [45]. During this period, CRC mortality gradually declined, especially in men. Although the authors concluded that this could be the result of an improved drinking water supply system, it must be noted that this change was likely multifactorial and that confounding factors were not integrated in the analyses.

Cancer incidence rates in Appalachian Kentucky are particularly concerning. A study compared Appalachian Kentucky counties—known for their exposure to coal contaminants including As—with control urban county [46]. A similar study conducted in China showed consistent results [23].

Measurement of As concentrations in toenail samples from 239 patients showed higher As levels as well as higher incidence and mortality rates related to CRC in the study group. Analyses revealed lower As concentrations in cancer as compared to non-cancer samples, with median values of 0.27 *vs.* 1.08 µg/kg, respectively (*p* = 0.007) [21].

### Synthesis of evidence

**Fig. 2:**
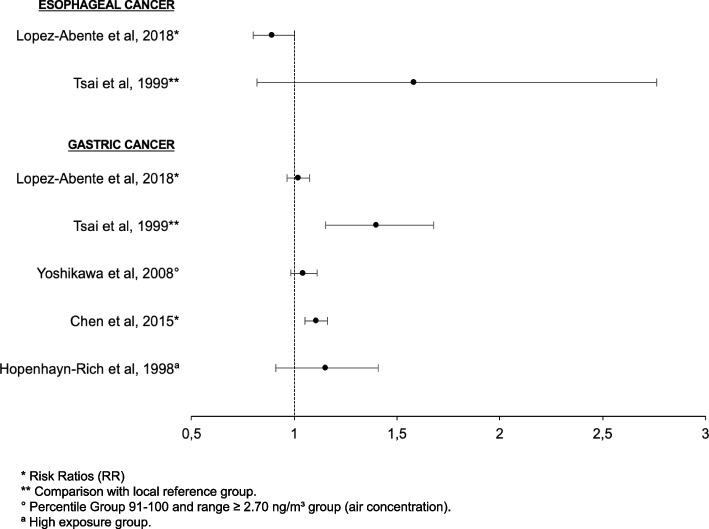
Associations between arsenic (As) and esogastric cancers. Forest plot illustrating the Standardized Mortality Rates (SMR) of esogastric cancers in women

**Fig. 3:**
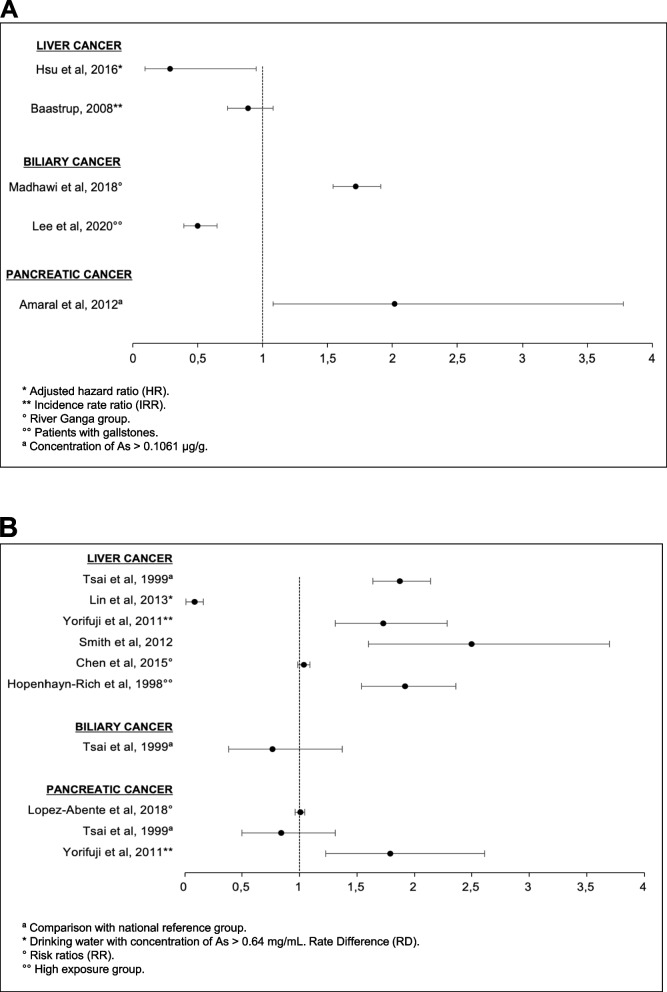
Associations between arsenic (As) and hepatopancreaticobiliary (HPB) cancers. **A** Forest plot illustrating Odds Ratios (OR) for the incidence of HPB cancers in men and women. **B** Forest plot illustrating the Standardized Mortality Rates (SMR) of HPB cancers in women, except for two studies that included women and men (Yorifuji et al. and Smith et al.)

**Fig. 4:**
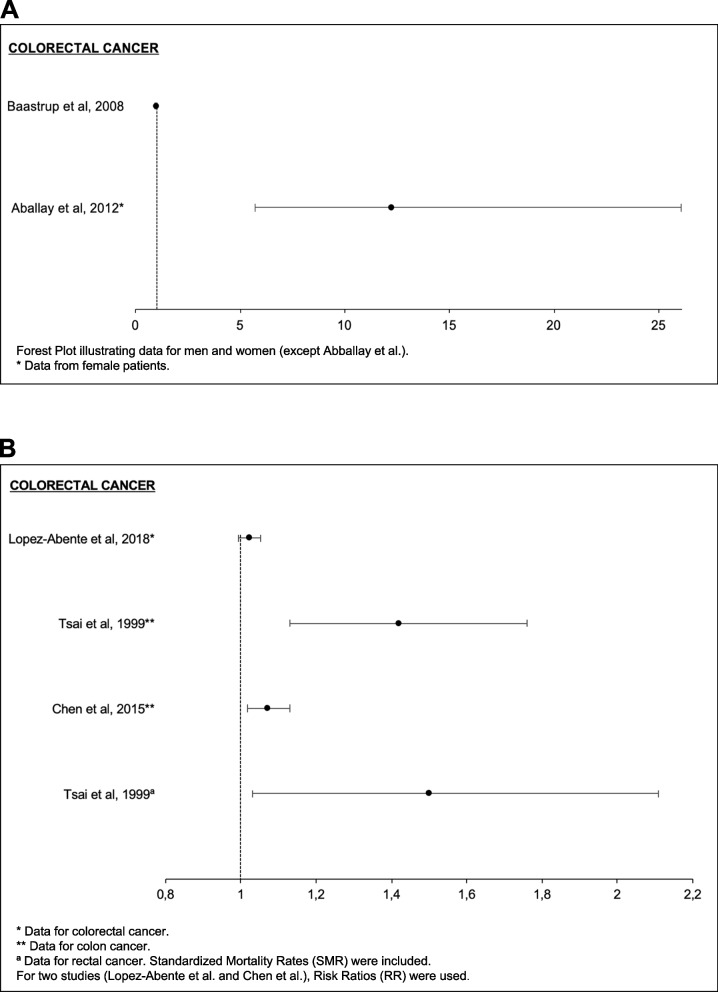
Associations between arsenic (As) and colorectal cancers. **A** Forest plot illustrating Odds Ratios (OR) for the incidence of colorectal cancers. **B** Forest plot illustrating the Standardized Mortality Rates (SMR) of colorectal cancers in women

The present systematic review identified studies exploring the potential association between As and digestive cancers with different perspectives and endpoints, focusing either on incidence or on mortality. Forest plots aimed to illustrate these associations. For esogastric cancers (Fig. 2), available studies investigated SMR. Among 7 studies, 2 articles identified an association. HPB cancers were the most frequently studied malignancies, with studies investigating their incidence and cancer-related mortality. A subset of 40% (2/5) and 50% (5/10) studies revealed association with As for incidence and mortality, respectively (Fig. 3). Finally, an association between As and colorectal cancers was detected for their incidence and mortality in 1/2 and 3/4 selected studies, respectively (Fig. 4). Overall, 43% (3/7) and 48% (10/21) studies highlighted an association between As and the incidence and the mortality of digestive cancers, respectively.

## Discussion

This study systematically reviewed the available data on the potential association between As and digestive cancers.

As is a documented carcinogen for bladder, lung and skin cancers [47]. These organs are major interfaces with our environment and the exposure to As through contaminated dust or direct contact is unsurprisingly associated with lung and skin cancers [48]. The association with urogenital cancers can be explained by the excretion of As metabolites in urine [49]. However, the lack of evidence for the carcinogenic effect of As on digestive organs is surprising. As is metabolized by the liver, generating MMA and DMA [50]. While MMA is mainly excreted in urine, DMA—the most toxic metabolite—is preferentially excreted in the bile. The digestive tract is thereby exposed to (I) iAs ingested in food and water, (II) primarily exposed through its metabolism (liver) and finally (III) exposed to its most toxic products that are released in the bile [11]. These considerations gave the impulsion to perform this systematic review.

This systematic review identified 35 studies. First, this is a very small number, considering that it explored all digestive cancers and that these are responsible for almost 5 million new cases each year [1]. In addition, most studies included in this review did not specifically aim at interrogating the role of As in a specific type of digestive cancer. In other words, these studies used large panels of various heavy metals—including As—and analyzed their association with a variety of health conditions, including digestive cancers. Therefore, an important degree of heterogeneity was observed among the selected studies. Interestingly, it seems that dedicated studies focusing on this potential link are progressively being published over the last years. Available data essentially derived from ecological, cohort and case–control studies. The resulting level of evidence is inherently limited. Nonetheless, the signal detected by the quantitative synthesis of the present systematic review showed that an important proportion of studies with 43% and 48% suggested an association between As and digestive cancers for their incidence and mortality, respectively. Analysis of these data showed that the proportion of studies demonstrating an association varied according to anatomical location. Even within the HPB group, liver and bile ducts seem more susceptible to the effect of As, compared to pancreas. This is in line with a metabolism-related hypothesis, suggesting that cells in contact with bile (into which the most toxic metabolites are excreted) are likely to experience chronic carcinogenic injury by DMA. Gallbladder, bile ducts and liver would be the principal targets of As-triggered carcinogenesis, followed by a declining trend for pancreas, small bowel, colon and rectum.

Some limitations need to be discussed. The main one is intrinsic to the topic rather than to the methodology of the present study: selected studies are few and of overall modest quality. In addition, they showed a high degree of heterogeneity for various aspects: study design, sample size, endpoints and technique of measurement of As concentrations. In addition, most studies failed to provide precise data regarding the types of cancers (e.g. most studies on LC only referred “liver cancer” but not specifically HCC or CCA). Finally, a meta-analysis was not feasible due to the different endpoints reported in the selected studies. Tackling the question of As in digestive cancers by a systematic review revealed evidence emphasizing the importance of further studies in this area. There is an urgent need to develop new strategies of prevention, which is likely the best—if not the only—way of controlling mortality related to these aggressive cancers (e.g. gallbladder adenocarcinoma). Mitigating As effects and controlling the levels of As on a large-scale is a challenging task, particularly in developing countries. As exposure includes multiple paths; if one only considers drinking water, WHO has set a precise threshold (i.e. 10 µg/L) and made clear recommendations [51]. The issue is that governmental agencies only control public wells but private wells remain an important source of drinking water in some regions and these are not monitored.

The present study also emphasizes specific points that need to be considered in future studies, like the importance to distinguish the varying effects according to the different species of As.

## Conclusions

This systematic review identified 35 studies that investigated the potential assocation of As on digestive cancers. Results suggest that As may be a determinant in digestive cancers, in particular in hepatobiliary cancers. It reinforces the rationale and underscores the need to conduct future studies focusing on this question to provide data of high quality on an individual basis. Documenting a carcinogenic effect of As in digestive cancers may have a substantial and potentially beneficial impact, especially in terms of prevention.

## Supplementary Information

**Additional file 1.** Search algorithms.

**Additional file 2.** Quality assessment of the selected studies.

**Additional file 3.** Selected studies investigating the effect of arsenic (As) in esogastric cancers.

**Additional file 4.** Selected studies investigating the effect of arsenic (As) in hepato-pancreatico-biliary cancers [52–54].

**Additional file 5.** Selected studies investigating the effect of arsenic (As) in colorectal cancers [55].

## Data Availability

Not applicable (data already publicly available).

## Abbreviations

aOR: Adjusted odds ratio
As: Arsenic
BDC: Bile duct cancer
BFD: Blackfoot-disease
CCA: Cholangiocarcinoma
CRC: Colorectal cancer
DMA: Dimethylarsonic acid
EC: Esophageal cancer
EPC: Exocrine pancreatic cancer
GBC: Gallbladder cancer
GC: Gastric cancer
HBV: Hepatitis-B virus
HCV: Hepatitis-C virus
HCC: Hepatocellular carcinoma
HPB: Hepatopancreaticobiliary
IRR: Incidence rate ratio
ICP-MS: Inductively coupled plasma mass spectrometry
LC: Liver cancer
MMA: Monomethylarsonic acid
MR: Mortality ratio
Ni: Nickel
OR: Odds ratio
PC: Pancreatic cancer
PDAC: Pancreatic ductal adenocarcinoma
RR: Risk ratio
SMR: Standardized mortality ratio
WHO: World Health Organization
