# Supplementary material for: Carcinogenic effect of arsenic in digestive cancers: a systematic review
Source: Environ Health. 2023 Apr 17;22:36. doi: 10.1186/s12940-023-00988-7 (PMC10108502; doi:10.1186/s12940-023-00988-7)
Supplement: Supplementary file 1 — Additional file 1. Search algorithms. [file 12940_2023_988_MOESM1_ESM.docx]

# **Additional File 1: Search algorithms**

# Medline Ovid SP

(Arsenic poisoning/ OR Arsenic/ OR Metalloids/ OR exp Arsenicals/ OR exp Arsenic Trioxide/ OR Arsenite Transporting ATPases/ OR Apatites/ OR (Arsenic* OR arsenous OR arsenite* OR arsenious OR arsenate* OR arsenolite OR arsentrioxide OR Arsanilic* OR Arsenide* OR Tetraarsenic* OR Tetra-arsenic OR trisenox OR Cacodylate OR arsenamid*).ab,ti,kf.) AND (exp Digestive System Neoplasms/ OR Neuroendocrine Tumors/ OR exp Carcinoid tumor/ OR exp "Carcinoma, Neuroendocrine"/ OR (((neoplasm* OR tumor* OR tumour* OR cancer* OR carcino* OR adenocarcino* OR sarcoma* OR adenom* OR oncolog* OR malignan* OR ampuloma* OR apudoma*) ADJ6 (esophag* OR intestin* OR cecal OR appendi* OR colorect* OR duoden* OR ileal OR jejun* OR stomach OR gastric OR gastro* OR colonic OR sigmoid OR colorectal OR rectal OR rectum OR anus OR anal OR liver OR hepatocellular OR hepato* OR hepatic* OR gallbladder OR 'bile duct*' OR bowel OR colon OR pancrea* OR 'alpha cell' OR 'beta cell' OR 'islet cell' OR peritoneal OR biliary OR digestive OR gut OR endocrine*)) OR nonpolyposis OR 'immunoproliferative small intestinal disease' OR somatostatinoma* OR vipoma* OR glucagonoma OR insulinoma OR insuloma).ab,ti,kf.)

# PubMed (Not Medline)

("Arsenic poisoning"[MeSH] OR "Arsenic"[MeSH] OR "Metalloids"[MeSH] OR "Arsenicals"[MeSH] OR "Arsenic Trioxide"[MeSH] OR "Arsenite Transporting ATPases"[MeSH] OR "Apatites"[MeSH] OR (Arsenic*[tiab] OR arsenous[tiab] OR arsenite*[tiab] OR arsenious[tiab] OR arsenate*[tiab] OR arsenolite[tiab] OR arsentrioxide[tiab] OR Arsanilic*[tiab] OR Arsenide*[tiab] OR Tetraarsenic*[tiab] OR Tetra-arsenic[tiab] OR trisenox[tiab] OR Cacodylate[tiab] OR arsenamid*[tiab])) AND ("Digestive System Neoplasms"[MeSH] OR "Neuroendocrine Tumors"[MeSH] OR "Carcinoid tumor"[MeSH] OR "Carcinoma, Neuroendocrine"[MeSH] OR (((neoplasm*[tiab] OR tumor*[tiab] OR tumour*[tiab] OR cancer*[tiab] OR carcino*[tiab] OR adenocarcino*[tiab] OR sarcoma*[tiab] OR adenom*[tiab] OR oncolog*[tiab] OR malignan*[tiab] OR ampuloma*[tiab] OR apudoma*) AND (esophag*[tiab] OR intestin*[tiab] OR cecal[tiab] OR appendi*[tiab] OR colorect*[tiab] OR duoden*[tiab] OR ileal[tiab] OR jejun*[tiab] OR stomach[tiab] OR gastric[tiab] OR gastro*[tiab] OR colonic[tiab] OR sigmoid[tiab] OR colorectal[tiab] OR rectal[tiab] OR rectum[tiab] OR anus[tiab] OR anal[tiab] OR liver[tiab] OR hepatocellular[tiab] OR hepato*[tiab] OR hepatic*[tiab] OR gallbladder[tiab] OR "bile duct*"[tiab] OR bowel[tiab] OR colon[tiab] OR pancrea*[tiab] OR "alpha cell*"[tiab] OR "beta cell*"[tiab] OR "islet cell*"[tiab] OR peritoneal[tiab] OR biliary[tiab] OR digestive[tiab] OR gut[tiab] OR endocrine*[tiab])) OR nonpolyposis[tiab] OR "immunoproliferative small intestinal disease"[tiab] OR somatostatinoma*[tiab] OR vipoma*[tiab] OR glucagonoma[tiab] OR insulinoma[tiab] OR insuloma[tiab])) NOT medline[sb]

# Embase.com

('arsenic poisoning'/de OR 'arsenic'/de OR 'arsenic derivative'/de OR 'organoarsenic derivative'/exp OR 'arsenic trioxide'/de OR 'arsenic acid'/de OR 'arsenic acid derivative'/de OR 'arsenous acid derivative'/de OR 'arsenate sodium'/de OR 'arsenite sodium'/de OR (Arsenic* OR arsenous OR arsenite* OR arsenious OR arsenate* OR arsenolite OR arsentrioxide OR Arsanilic* OR Arsenide* OR Tetraarsenic* OR Tetra-arsenic OR trisenox OR Cacodylate OR arsenamid*):ab,ti,kw) AND ('digestive system tumor'/exp OR 'digestive system tumor cell line'/exp OR 'gastrointestinal carcinoid'/exp OR 'gastroenteropancreatic neuroendocrine tumor'/exp OR (((neoplasm* OR tumor* OR tumour* OR cancer* OR carcino* OR adenocarcino* OR sarcoma* OR adenom* OR oncolog* OR malignan* OR ampuloma* OR apudoma*) NEAR/6 (esophag* OR intestin* OR cecal OR appendi* OR colorect* OR duoden* OR ileal OR jejun* OR stomach OR gastric OR gastro* OR colonic OR sigmoid OR colorectal OR rectal OR rectum OR anus OR anal OR liver OR hepatocellular OR hepato* OR hepatic* OR gallbladder OR 'bile duct*' OR bowel OR colon OR pancrea* OR 'alpha cell' OR 'beta cell' OR 'islet cell' OR peritoneal OR biliary OR digestive OR gut OR endocrin*)):ab,ti,kw) OR (nonpolyposis OR 'immunoproliferative small intestinal disease' OR somatostatinoma* OR vipoma* OR glucagonoma OR insulinoma OR insuloma OR nonpolyposis):ab,ti,kw)

# Cochrane Library

(arseni* OR arsenat* OR arsenolite* OR arsentrioxide OR organoarsenic OR arsine* OR arsenous):ab,ti,kw AND ((((neoplasm* OR tumor* OR tumour* OR cancer* OR carcino* OR adenocarcino* OR sarcoma* OR adenom* OR oncolog* OR malignan* OR ampuloma* OR apudoma*) NEAR/6 (esophag* OR intestin* OR cecal OR appendi* OR colorect* OR duoden* OR ileal OR jejun* OR stomach OR gastric OR gastro* OR colonic OR sigmoid OR colorectal OR rectal OR rectum OR anus OR anal OR liver OR hepatocellular OR hepato* OR hepatic* OR gallbladder OR 'bile duct*' OR bowel OR colon OR pancrea* OR 'alpha cell' OR 'beta cell' OR 'islet cell' OR peritoneal OR biliary OR digestive OR gut OR endocrin*)):ab,ti,kw) OR (nonpolyposis OR 'immunoproliferative small intestinal disease' OR somatostatinoma* OR vipoma* OR glucagonoma OR insulinoma OR insuloma OR nonpolyposis):ab,ti,kw)

# Web of Science Core Collection

TS=((Arsenic* OR arsenous OR arsenite* OR arsenious OR arsenate* OR arsenolite OR arsentrioxide OR Arsanilic* OR Arsenide* OR Tetraarsenic* OR Tetra-arsenic OR trisenox OR Cacodylate OR arsenamid*) AND (((neoplasm* OR tumor* OR tumour* OR cancer* OR carcino* OR adenocarcino* OR sarcoma* OR adenom* OR oncolog* OR malignan* OR ampuloma* OR apudoma*) NEAR/6 (esophag* OR intestin* OR cecal OR appendi* OR colorect* OR duoden* OR ileal OR jejun* OR stomach OR gastric OR gastro* OR colonic OR sigmoid OR colorectal OR rectal OR rectum OR anus OR anal OR liver OR hepatocellular OR hepato* OR hepatic* OR gallbladder OR "bile duct*" OR bowel OR colon OR pancrea* OR "alpha cell" OR "beta cell" OR "islet cell" OR peritoneal OR biliary OR digestive OR gut OR endocrin*)) OR (nonpolyposis OR "immunoproliferative small intestinal disease" OR somatostatinoma* OR vipoma* OR glucagonoma OR insulinoma OR insuloma OR nonpolyposis)))

# Supplementary search

## Google Scholar

Arsenic|arsenous|arsenite|arsenious|arsenate|arsenolite|arsentrioxide|Arsanilic Gastrointestinal|Esophagus|intestine|digestive|liver|gallbladder|pancreatic|colon|endocrine|gastric|hepatocellular neoplasm|tumor|tumour|cancer|carcinoma|sarcoma|adenoma|ampuloma
