# Supplementary material for: Carcinogenic effect of arsenic in digestive cancers: a systematic review
Source: Environ Health. 2023 Apr 17;22:36. doi: 10.1186/s12940-023-00988-7 (PMC10108502; doi:10.1186/s12940-023-00988-7)
Supplement: Supplementary file 2 — Additional file 2. Quality assessment of the selected studies. [file 12940_2023_988_MOESM2_ESM.docx]

**Additional File 2: Quality assessment of the selected studies**

|  | | **Quality criteria** | | | | | | |
| --- | --- | --- | --- | --- | --- | --- | --- | --- |
| **First author, Year** | **Study design** | Selection and attrition bias | Exposure misclassification | Outcome missclassification | Potential confouding | Selective reporting | Analysis | Study sensitivity |
| Ganesan, 2020 | Ecological | + | + / ↓ | ++ / ↓ | ++ | +++ | ++ | +++ |
| Reyes, 2022 | Ecological | + | + / ↓ | ++ / ↓ | ++ | +++ | ++ | +++ |
| Lopez-Abente, 2018 | Ecological | + | + / ↓ | + / ↓ | +++ | +++ | +++ | ++ |
| Lee, 2016 | Ecological | + | + / ↓ | ++ | 0 | ++ | ++ | ++ |
| Tsai, 1999 | Ecological | + | + / ↓ | ++ | ++ | +++ | ++ | ++ |
| Kazi, 2015 | Case-control | ++ / ↑ | ++ / ↓ | +++ | ++ | +++ | ++ | ++ |
| Nozadi, 2021 | Case-control | ++ / ↑ | +++ | +++ | +++ | ++ | ++ | ++ |
| Kumar, 2021 | Case-control | ++ / ↑ | +++ | +++ | + | +++ | + | ++ |
| Yoshikawa, 2008 | Ecological | + | + / ↓ | + / ↓ | ++ | +++ | ++ | ++ |
| Chen, 2015 | Ecological | + | ++ / ↓ | ++ | +++ | +++ | ++ | ++ |
| Kohzadi, 2017 | Case-control | ++ / ↑ | +++ | +++ | + | +++ | + | + |
| McKinley, 2013 | Ecological | + / ↑ | ++ / ↓ | +++ | + | +++ | ++ | +++ |
| Hopenhayn-Rich, 1998 | Ecological | + | ++ / ↓ | ++ / ↑ | +++ | +++ | ++ | +++ |
| Chen, 1986 | Case-control | ++ / ↑ | + / ↓ | +++ | ++ | +++ | ++ | ++ |
| Guo, 2003 | Ecological | + / ↑ | + / ↓ | +++ | + | +++ | + | + |
| Lin, 2013 | Ecological | + | + / ↓ | + / ↓ | + | +++ | ++ | ++ |
| Yorifuji, 2011 | Case-control | ++ / ↑ | ++ / ↓ | ++/ ↓ | 0 | +++ | + | ++ |
| Smith, 2012 | Ecological | + / ↑ | + / ↓ | +/ ↓ | + | +++ | + | ++ |
| Baastrup, 2008 | Cohort | +++ | +++ | +++ | +++ | +++ | +++ | +++ |
| Elwakil,2017 | Case-control | ++ / ↑ | +++ | +++ | 0 | +++ | ++ | ++ |
| Cano, 2021 | Case-control | ++ / ↑ | +++ | +++ | 0 | +++ | +++ | ++ |
| Chen, 2004 | Cohort | ++ / ↑ | ++ / ↓ | +/ ↓ | 0 | +++ | + | ++ |
| Hsu, 2016 | Cohort | ++ / ↑ | +++ | +++ | + | +++ | ++ | ++ |
| Madhawi, 2018 | Cohort | ++ / ↑ | ++ / ↓ | +++ | 0 | +++ | + | + |
| Lee, 2020 | Case-control | ++ / ↑ | +++ | +++ | +++ | +++ | +++ | +++ |
| Barahona Ponce, 2020 | Cohort | ++ / ↑ | + / ↓ | +++ | ++ | ++ | ++ | ++ |
| Amaral, 2012 | Case-control | +++ | +++ | +++ | +++ | +++ | ++ | +++ |
| Gomez-Tomas, 2019 | Case-control | ++ / ↑ | ++ / ↓ | +++ | ++ | +++ | ++ | +++ |
| Abballay, 2012 | Ecological | + | ++ / ↓ | +++ | ++ | +++ | +++ | +++ |
| Yang, 2008 | Ecological | + | ++ | +++ | 0 | ++ | + | + |
| Johnson, 2011 | Case-control | ++ / ↑ | ++ / ↓ | ++/ ↓ | 0 | ++ | + | + |
| Cicalese, 2017 | Ecological | + | + / ↓ | ++/ ↓ | + | +++ | + | + |
| Han, 2009 | Ecological | + | + / ↓ | ++/ ↓ | ++ | +++ | ++ | ++ |
| Wu, 2021 | Ecological | + | + | ++/ ↓ | + | +++ | ++ | ++ |
| Helmfrid, 2019 | Case-control | ++ / ↑ | +++ | +++ | +++ | +++ | +++ | +++ |
