# Supplementary material for: Carcinogenic effect of arsenic in digestive cancers: a systematic review
Source: Environ Health. 2023 Apr 17;22:36. doi: 10.1186/s12940-023-00988-7 (PMC10108502; doi:10.1186/s12940-023-00988-7)
Supplement: Supplementary file 3 — Additional file 3. Selected studies investigating the effect of arsenic (As) in esogastric cancers. [file 12940_2023_988_MOESM3_ESM.docx]

**Additional File 3: Selected studies investigating the effect of arsenic (As) in esogastric cancers**

| **Design / Year of publication** | **Country** | **Number of patients** | **As exposure** | **Summary and main findings** | **Ref** |
| --- | --- | --- | --- | --- | --- |
| **ESOPHAGUS** | | | | |  |
| Ecological / 2018 | Spain | EC: 16,515 deaths  Total: 861,440 | Topsoil | As was associated with a lower mortality in women with EC (RR: 0.89 95% CI: 0.80-1.00). No effect was detected in men. | [17] |
| Ecological / 2016 | Taiwan | 209 | Topsoil | As in farm soils did not correlate with the prevalence of EC. | [18] |
| Ecological / 1999 | Taiwan | EC: 81  Total CD: 20,067 | Water | In BFD endemic area, EC mortality in men was higher than in control regions (SMR: 1.67, 95% CI 1.30-2.12). No difference was observed in women. | [19] |
| Case-control / 2015 | Pakistan | 126 | NA | Higher concentrations of As were measured in male patients with EC, compared to healthy controls. | [20] |
| Case-control / 2021 | Iran | EC : 19  Controls : 63 | NA | As was measured in EC tissue samples and compared to non-cancer samples.  Median concentrations were comparable in both groups. | [21] |
| Case-control / 2021 | India | EC: 18  Controls: 200 | NA | No conclusion specific to EC | [40] |
| **STOMACH** | | | | |  |
| Ecological / 2018 | Spain | GC: 56,371 deaths  Total: 861,440 | Topsoil | As was not associated with a GC mortality. | [17] |
| Ecological / 2008 | Japan | 126,697 | Air | No effect of As concentrations on GC mortality. | [22] |
| Ecological / 2015 | China | 46’675 deaths  GC: 14,022 deaths | Soil | Correlation between As in soil and age-adjusted cancer-related mortality  Spearman=0.412, p<0.01  ♂: RR: 1.114, 95% CI: 1.063-1.168, p<0.001  ♀: RR: 1.105, 95% CI 1.051-1.161, p<0.001 | [23] |
| Case-control / 2017 | Iran | GC: 35  Controls: 30 | N/A | Higher level of As in tissue samples from cancer patients (cancer and non-cancer samples) compared to controls. | [24] |
| Ecological / 2013 | Ireland | N/A | Soil | Correlation between GC incidence and As level showed a wide range of values. | [25] |
| Ecological / 1998 | Argentina | N/A | Water | No effect of As on GC-related mortality.  ♂: SMR: 1.04, 95% CI: 0.87-1.22  ♀: SMR: 1.15, 95% CI 0.91-1.41 | [26] |
| Ecological / 1999 | Taiwan | PC: 306  Total CD: 20,067 | Water | In BFD endemic area, GC mortality was higher in men (SMR: 1.36, 95% CI 1.17-1.46) and in women (SMR: 1.40, 95% CI 1.15-1.68), than in control regions | [19] |
| Case-control / 2021 | Iran | GC: 20  Controls: 63 | NA | As was measured in GC tissue samples and compared to non-cancer samples.  As levels in tissue of GC and control patients were comparable. | [21] |
| Case-control / 2021 | India | GC: 90  Controls: 200 | NA | No conclusion specific to GC. | [40] |

As: arsenic ; BFD : blackfoot disease ; CI : confidence interval ; EC: esophageal cancer ; GC : gastric cancer ; N/A : Not applicable ; NA : not available ; OR : odd ratio ; RR : relative risk ; SMR : standard mortality ratio
