# Supplementary material for: Carcinogenic effect of arsenic in digestive cancers: a systematic review
Source: Environ Health. 2023 Apr 17;22:36. doi: 10.1186/s12940-023-00988-7 (PMC10108502; doi:10.1186/s12940-023-00988-7)
Supplement: Supplementary file 4 — Additional file 4. Selected studies investigating the effect of arsenic (As) in hepato-pancreatico-biliary cancers [52–54]. [file 12940_2023_988_MOESM4_ESM.docx]

**Additional File 4: Selected studies investigating the effect of arsenic (As) in hepato-pancreatico-biliary cancers**

| **Design / Year of publication** | **Regions** | **Number of patients** | **As exposure** | **Summary and main findings** | **Ref** |
| --- | --- | --- | --- | --- | --- |
| **LIVER** | | | | | |
| Ecological / 1999 | Taiwan | LC: 855  Total CD: 20,067 | Water | In BFD endemic area, LC mortality was higher than in men (SMR: 1.83, 95% CI 1.69-1.98) and in women (SMR: 1.87, 95% CI 1.64-2.14), compared to control regions. | [19] |
| Case-control / 1986 | Taiwan | LC: 59  Controls: 368 | Water | Incidence of LC in BFD areas was compared to population not exposed to high level of As in water. Individuals from BFD areas exposed to As drinking water over 40 years showed higher risk to develop LC (OR: 2.67, p<0.01). | [27] |
| Ecological / 2003 | Taiwan | 40'832 | Water | Incidence of HCC and CCA were comparable in BFD and control regions. | [28] |
| Ecological / 2013 | Taiwan | LC: 1003 | Water | As level > 0.64 mg/L was associated with higher LC-related mortality in men (RD: 0.22, 95% CI 0.06-0.38, p<0.01) and in women (RD: 0.09, 95% CI 0.01-0.16, p<0.05). Concentrations <0.64 mg/L were not associated with deleterious impact. | [29] |
| Cohort /  2016 | Taiwan | 8086 | Water | Seropositive patients for HBsAg or anti-HCV who consumed water with levels of As ≥100.0 μg/L had reduced risk of liver cancer | [36] |
| Case-control / 2011 | Japan | LC: 153  Total: 3141 | Milk powder | A mass As poisoning occurred in Western Japan in 1955. Comparison between the exposed and non-exposed cohorts suggested a carcinogenic effect of As in LC (MR: 1.73, 95% CI: 1.31-2.28). | [30] |
| Ecological / 2012 | Chile | LC : 25 | Water | The city of Antofagasta in Chile was exposed to high level of As in water between 1958-1070.  LC-related mortality of the exposed population was higher than in the rest of Chile (SMR: 2.5; 95% CI: 1.6-3.7). | [31] |
| Ecological / 2017 | USA | 43,144 | Air | As-polluted air was inconsistently associated with HCC incidence in Texas. | [52] |
| Ecological / 2015 | China | 46’675 deaths  LC: 10,076 deaths | Soil | Correlation between As in soil and age-adjusted cancer-related mortality  Spearman=0.174, p: NS  ♂: RR: 1.032, 95% CI: 0.988-1.078, p=0.158  ♀: RR: 1.037, 95% CI: 0.986-1.090, p=0.160 | [23] |
| Cohort /  2004 | Bangladesh | 65,876 individuals | Water | Lifetime excess risk attributable to As in drinking water for LC was 0.9 in men and 3.4 in women, per 100,000 population. | [35] |
| Case-control /  2017 | Egypt | 314 | Water | As blood concentration was significantly higher in HCC patients compared to control subjects | [33] |
| Ecological / 1998 | Argentina | N/A | Water | Exposure to high level of As was associated with higher SMR.  ♂: SMR: 1.84, 95% CI: 1.49-2.24  ♀: SMR: 1.92, 95% CI 1.54-2.36 | [26] |
| Ecological /  2009 | USA | N/A | Water | Exposure to low level As in ground water is not associated with liver cancer incidence | [53] |
| Cohort /  2008 | Denmark | LC: 35  Total: 57,053 | Water | No significant association between exposure to As and incidence of liver cancer (LC) (IRR: 0.89, 95% CI: 0.73-1.08, p=0.24). | [32] |
| Ecological / 2021 | India | N/A | Water | Geostatistical model of As distribution to infer As-induced LC. The model suggested that As level >10 µg/L in groundwater was not associated with increased rate of LC. | [54] |
| Case-control / 2021 | Peru France | 76 HCC | NA | As was measured in tumor and non-tumor tissue of two cohorts of patients developing HCC on non-cirrhotic liver (Peru and France). Higher As levels were detected in Peru compared to French cohort, both in tumor and non-tumoral tissues. Of note, As was detected above the quantification limit only in a subset of Peruvian samples. | [34] |
| Case-control / 2021 | India | GBC: 118  Controls: 200 | NA | Significant correlation between blood As level and age of LC patients | [40] |
| **BILIARY** | | | | | |
| Ecological / 1999 | Taiwan | Total CD: 20,067  GBC: 24 | Water | In BFD endemic area, GBC mortality was comparable to control regions, both for men and women. | [19] |
| Ecological / 2020 | Worlwide  USA  Taiwan  India | NA | Water | Worldwide:  ♀: Spearman=0.31, p=0.03  ♂Spearman=0.06, p=0.66  USA:  ♀: Spearman=0.05, p=0.42  ♂Spearman=0.14, p=0.03  Taiwan:  ♀: Spearman=0.57, p<0.01  ♂Spearman=0.38, p=0.08  India:  Regions with high-level of As (>50µg/L) showed higher incidence of ECC in women (R^2^=0.23, p<0.01) and men (R^2^=0.26, p<0.01) | [8] |
| Cohort / 2018 | India | 1291 | NA | Higher rates of GBC were reported in the districts along main central River Ganga (OR: 1.72, 95% CI: 1.54-1.91, p=0.001) and those exposed to high As soil content (OR: 1.45, 95% CI: 1.30-1.62, p=0.001). | [37] |
| Case-control / 2020 | China | GBC: 259  GS: 701  Controls: 851 | NA | Levels of As in serum of GBC patients were lower than patients with gallstones or healthy controls. Regardless of gallstones, As was associated with a reduced odds ratio for GBC: without gallstones (OR: 0.2, 95% CI: 0.13-0.29, p<0.001), with gallstones (OR: 0.50, 95% CI: 0.39-0.65, p<0.001) (highest tertile). | [38] |
| Cohort / 2020 | Europe | N/A | NA | Mendelian randomization to assess the causal effect of As species in GBC; iAs% (OR: 0.80, p=0.03) and MMA% (OR: 0.85, p=0.08) had a protective effect while DMA% (OR: 1.10, p=0.06) showed a deleterious impact on GBC | [39] |
| Ecological / 2022 | USA  Taiwan  India | NA | Water | USA:  As had no effect on both intrahepatic- (ICC) and extrahepatic- cholangiocarcinoma (ECC) incidence  Taiwan:  *ICC*  ♀: Spearman=0.67, p<0.01  ♂Spearman=0.55, p=0.01  *ECC*  ♀: Spearman=0.20, p=0.375  ♂Spearman=0.62, p=0.003  India:  Regions with high-level of As (>50µg/L) showed higher incidence of ECC in women (R^2^=0.31, p<0.01) and men (R^2^=0.26, p=0.01) | [9] |
| Case-control / 2021 | India | GBC: 175  Controls: 200 | NA | Significant correlation between blood As level and age of GBC patients | [40] |
| **PANCREAS** | | | | | |
| Ecological / 2018 | Spain | PC: 42,274 deaths  Total: 861,440 | Topsoil | As was not associated with PC-related mortality, both in men and in women | [17] |
| Ecological/  1999 | Taiwan | PC: 49  Total CD: 20,067 | Water | In BFD endemic area, PC mortality was comparable to local reference in men and women. | [19] |
| Case-control/ 2011 | Japan | PC: 87  Total: 3141 | Milk powder | A mass As poisoning occurred in Western Japan in 1955. Comparison between the exposed and non-exposed cohorts suggested a carcinogenic effect of As in PC (MR: 1.79, 95% CI: 1.23-2.61). | [30] |
| Case-control / 2012 | Spain | EPC: 118  Controls: 399 | NA | Analysis of trace elements including As in toenails of patients with exocrine pancreatic cancer (EPC) and healthy controls. Patients in the highest quartile for As level were at higher risk to develop EPC (OR: 2.02, 95% CI: 1.08-3.78, p=0.009). | [42] |
| Case-control / 2019 | Spain | PDAC: 78  Controls: 416 | NA | Analysis of trace elements including As in toenails of patients with pancreatic ductal adenocarcinoma (PDAC) and healthy controls. Correlation analyses between trace elements and KRAS mutation.  Higher level of As (higher tertile) was not associated with a significant risk of KRAS wild-type (aOR: 3.37, 95% CI: 0.98-11.57, p=0.067) or KRAS mutated (aOR: 1.73, 95% CI: 0.85-3.53, p=0.127) PDAC | [43] |

As: arsenic ; BFD : blackfoot disease ; CI : confidence interval ; GBC : gallbladder cancer ; ECC : extrahepatic- cholangiocarcinoma ; iAs : ionized arsenic ; ICC : intrahepatic- cholangiocarcinoma ; PC : pancreatic cancer ; PDAC : pancreatic ductal adenocarcinoma ; N/A : Not applicable ; NA : not available ; OR : odd ratio ; RR : relative risk ; SMR : standard mortality ratio
