# Supplementary material for: Carcinogenic effect of arsenic in digestive cancers: a systematic review
Source: Environ Health. 2023 Apr 17;22:36. doi: 10.1186/s12940-023-00988-7 (PMC10108502; doi:10.1186/s12940-023-00988-7)
Supplement: Supplementary file 5 — Additional file 5. Selected studies investigating the effect of arsenic (As) in colorectal cancers [55]. [file 12940_2023_988_MOESM5_ESM.docx]

**Additional File 5: Selected studies investigating the effect of arsenic (As) in colorectal cancers**

| **Design / Year of publication** | **Country** | **Number of patients** | **As exposure** | **Summary and main findings** | **Ref** |
| --- | --- | --- | --- | --- | --- |
| Cohort / 2008 | Denmark | CRC: 441  Total: 57,053 | Water | No significant association between exposure to low concentrations of As and incidence of CRC (IRR: 0.97, 95% CI: 0.93-1.01, p=0.1) | [32] |
| Ecological / 2018 | Spain | CRC: 119,663  Total: 861,440 | Topsoil | As was not associated with CRC-related mortality, both in men and in women | [17] |
| Ecological / 2012 | Argentina | N/A | Water | As in drinking water was associated with an increased incidence of CRC in women (IRR: 12.21, 95% CI: 5.72-26.07, p<0.01) and a decreased incidence in men (IRR: 0.03, 95% CI: 0.02-0.06, p<0.01). | [44] |
| Ecological / 1999 | Taiwan | Intestine: 23  Colon: 174  Rectum: 79  Total CD: 20,067 | Water | In BFD endemic area, CRC mortality was higher than in control regions, both in men and women.  Colon:  ♂: SMR: 1.49, 95% CI: 1.20-1.83  ♀: SMR: SMR: 1.42, 95% CI 1.13-1.76  Rectum:  ♂: SMR: 1.44, 95% CI: 1.05-1.92  ♀: SMR: SMR: 1.50, 95% CI 1.03-2.11 | [19] |
| Ecological / 2008 | Taiwan | CRC: 365 | Water | CRC-SMR declined between 1971 and 2006 in BDF-endemic area, especially in men. Authors attributed this change to the drinking-water supply system, which was improved in the early 1960s. | [45] |
| Case-control / 2011 | USA | 239 | Air, soil | Appalachian Kentucky is exposed to As. It was compared to control region, using toenail samples. Patients of the study group showed higher levels of As as well as higher CRC incidence and mortality rates. | [46] |
| Ecological /  2015 | China | 46’675 deaths  CC: 4367 deaths | Soil | Correlation between As in soil and age-adjusted cancer-related mortality  Spearman=0.369, p<0.01  ♂: RR: 1.094, 95% CI: 1.032-1.159, p=0.003  ♀: RR: SMR: 1.072, 95% CI 1.017-1.131, p=0.012 | [23] |
| Case-control / 2021 | Iran | CC: 24  Controls: 63 | NA | As was measured in CRC tissue samples and compared to non-cancer samples.  Controls showed higher concentration of As, compared to CRC patients. | [21] |
| Case-control / 2021 | India | CC: 21  RC: 29  Controls: 200 | NA | No conclusion specific to CRC nor RC. | [40] |
| Case-control /  2019 | Sweden | Total : 34’266  Questionnaire : 1846  RC : 17 | Local food, occupational exposure | High consumption of local food from glasswork contaminated area was not associated with higher concentration of As in blood and urine. Impact of As on CRC was not analyzed. | [55] |

As: arsenic ; BFD : blackfoot disease ; CI : confidence interval ; CRC : colorectal cancer ; N/A : Not applicable ; NA : not available ; OR : odd ratio ; RC : rectal cancer ; RR : relative risk ; SMR : standard mortality ratio
